# Prevalence and clinical characteristics of wheezing in children in the first year of life, living in Cuiabá, Mato Grosso, Brazil☆

**DOI:** 10.1016/j.rpped.2014.06.004

**Published:** 2014-12

**Authors:** Lillian Sanchez Lacerda Moraes, Olga Akiko Takano, Javier Mallol, Dirceu Solé

**Affiliations:** a Universidade Federal de Mato Grosso (UFMT), Cuiabá, MT, Brazil; b Universidade de Santiago, Santiago, Chile; c Universidade Federal de São Paulo (UNIFESP), São Paulo, SP, Brazil

**Keywords:** Infant, Respiratory sounds, Asthma, Prevalence

## Abstract

**OBJECTIVE:**

To evaluate the prevalence and the clinical characteristics of wheezing in infants aged 12 to 15 months in the city of Cuiabá, Mato Grosso State, Midwest Brazil.

**METHODS:**

Parents and/or guardians of infants were interviewed and completed a written standardized questionnaire of the Estudio Internacional de Sibilancia en Lactantes (EISL) - phase 3 at primary healthcare clinics at the same day of children vaccination or at home, from August of 2009 to November of 2010.

**RESULTS:**

1,060 parents and/or guardians completed the questionnaire, and 514 (48.5%) infants were male. Among the studied infants, 294 (27.7%) had at least one episode of wheezing during the first year of life, beggining at 5.8±3.0 months of age, with a predominance of male patients. The prevalence of occasional wheezing (<3 episodes of wheezing) was 15.0% and recurrent wheezing (≥3 episodes) was 12.7%. Among the infants with recurrent wheezing, the use of inhaled β2-agonist, oral corticosteroid, leukotriene receptor antagonist, as well as night symptoms, respiratory distress, and hospitalization due to severe episodes were significantly more frequent. Physician-diagnosed asthma was observed in 28 (9.5%) of the wheezing infants. Among the wheezing infants, 80 (27.7%) were diagnosed with pneumonia, of whom 33 (11.2%) required hospitalization; neverthless, no differences between occasional and recurrent wheezing infants were found.

**CONCLUSIONS:**

The prevalence of recurrent wheezing and physician-diagnosed asthma in infants were lower compared with those observed in other Brazilian studies. Recurrent wheezing had early onset and high morbity.

## Introduction

Wheezing is one of the most common respiratory symptoms in childhood and can manifest in several respiratory diseases; asthma is the most common. It is estimated that approximately 50% to 80% of children with asthma develop symptoms in the first five years of life, but diagnosis is difficult in this age group due to the difficulties in performing pulmonary function tests and the high prevalence of other causes of wheezing.^1^

Despite the significant impact of recurrent wheezing in childhood on public health, especially in developing countries, until recently there was no international comparative information on the prevalence of wheezing obtained by a standardized and validated tool, especially in the first year of life, when children are more vulnerable to complications from anatomical, functional, and immunological characteristics of the airways.^2^

To assess the impact of recurrent wheezing in infants in the first year of life and determine its prevalence and associated risk factors, the international study Estudio Internacional de Sibilancias en Lactantes (EISL) was developed. This is an international multicenter study comprising Latin America countries, Spain, and the Netherlands.^3^

The first data obtained in Brazil with the EISL were from 3,003 infants in the city of Curitiba, PR; it was observed that, in the first 12 months of life, 45.4% had at least one episode and 22.6% had recurrent wheezing episodes (three or more), evidencing a high prevalence of wheezing, with early onset and high morbidity.^4^ In São Paulo, SP, Brazil, in 1,014 infants living in the central-south region, 46% had at least one episode and 26.6% recurrent wheezing episodes. Also in São Paulo, the onset of wheezing was early, around five months of life, and the proportion of infants diagnosed and treated as asthmatics was low, which demonstrates the difficulty of attaining physician-diagnosed asthma in this age group.^5^

The prevalence of at least one episode of wheezing in the first year of life was 61% of infants residing in the city of Porto Alegre, RS, Brazil^6^ and 43% of infants in the city of Recife, PE, Brazil.^7^

The data obtained at the first phase of the study conducted in Latin America and Europe found that, of 30,093 infants, 45.2% had at least one episode and 20.3% had recurrent wheezing episodes, with a mean prevalence of recurrent wheezing in Latin American countries of 21.4%, while in European countries it was 15%. There was significant morbidity associated with recurrent wheezing in terms of severe episodes, visits to emergency rooms, hospital admissions, and use of inhaled corticosteroids.^8^

Considering that wheezing in infants is a very common symptom, whose prevalence varies in different centers, that the causes for such variations are still under investigation, and that Brazil is a country with a large territory with climatic, cultural, and socioeconomic differences, this study aimed to determine the prevalence and clinical characteristics of wheezing in infants living in Cuiabá, MT, using the standardized tool EISL - phase 3.

## Method

Parents or guardians of infants aged between 12 and 15 months living in the city of Cuiabá, state of Mato Grosso, Brazil, and who answered the standard EISL written questionnaire (WQ-EISL) - Phase 3 participated in this study. Parents and/or guardians were invited to participate in the study when upon seeking the Basic Health Units (BHU) for consultations and/or routine vaccination of their children or through home visits to children enrolled in the Family Health Program of the BHUs. This visit was conducted together with health care providers to facilitate access to households.

At the time when study was performed, there were 60 BHUs distributed in four regions (North, South, East, and West). In the North, West, and East regions, population density is medium-high: 57.4 to 86.0 inhabitants/hectare (inhab/ha); in the south region, population density is medium-low (11.05 to 28.76 inhab/ha).

Twenty-eight BHUs were selected by drawing lots to apply the written questionnaires, with six units in the North, West, and East regions and ten units in the South region, due to the lower population density. Visits to the BHUs occurred between August of 2009 and November of 2010 and during the two immunization campaigns against childhood polio conducted during this period; all children of the desired age group who came to the BHU on the day of the campaign participated in the study. Parents and/or guardians signed an informed consent form (ICF) and were then interviewed by the main researcher or by a previously trained medical student from Universidade Federal do Mato Grosso (UFMT).

The WQ-EISL phase 3 consists of 50 questions on demographic characteristics, wheezing, and risk factors; it has been translated into Brazilian Portuguese and culturally validated.^9^ For that study, it was determined by the coordinators of the EISL that the sample should include at least 1,000 infants. This sample size was based on the International Study of Asthma and Allergies in Childhood (ISAAC), considering a prevalence of wheezing of 30% and 25% in two different centers, with a power of study of 95% and a significance level of 1% for this sample, to ensure adequate power for comparisons between centers and countries, even for questions with a low prevalence of positive answers.^3^^,^ 10

Data were encoded in a standardized manner, transferred to a database developed in Microsoft Excel(r) 2007, and statistically analyzed using SPSS for Windows - release 18.0. Wheezing infants were separated into groups taking into account the frequency of wheezing: less than three episodes of wheezing (occasional wheezing) and three or more episodes of wheezing (recurrent wheezing). Those who had never had wheezing episodes were called non-wheezers. 

To analyze variable dependence, parametric (Student's *t*-test) and nonparametric (chi-squared and Fisher's exact test) tests were employed, as well as logistic regression for joint analysis of the possible factors related to the severity of recurrent wheezing. The significance level was set at 0.05 for the rejection of the null hypothesis.

This study was approved by the Research Ethics Committee of the Universidade Federal de São Paulo/Escola Paulista de Medicina - UNIFESP/EPM and by the Research Ethics Committee of the Hospital Universitário Júlio Müller/UFMT in Cuiabá-MT.

## Results

A total of 1,060 parents and/or guardians of infants aged between 12 and 15 months were interviewed. No questionnaire was excluded from the sample for being incorrectly filled out. Most respondents were mothers (87.9%), followed by other relatives (6.4%), and fathers (5.7%). Of the 1,060 children, 546 were females (51.5%). The general characteristics of the study population are shown in Table 1.

**Table 1:**
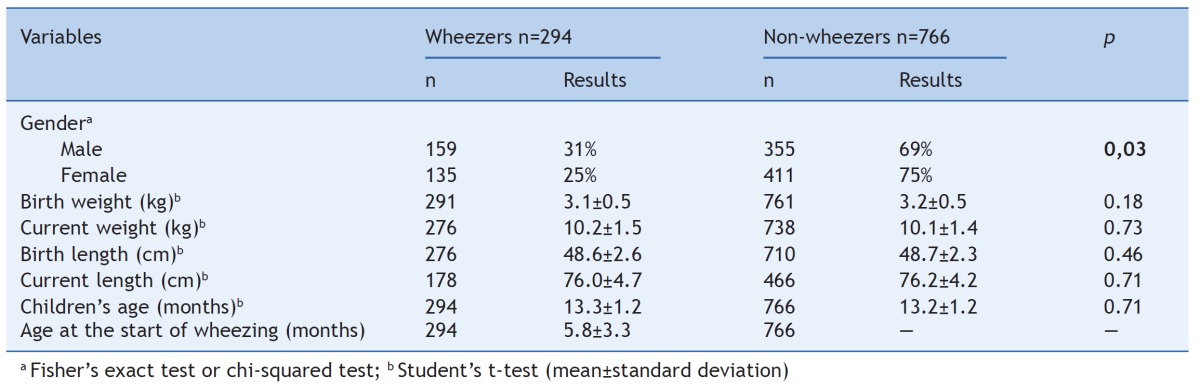
General characteristics of the study population (n=1,060).

Of the assessed infants, 294 (27.7%) had at least one episode of wheezing in the first year of life (wheezers), with wheezing onset at 5.8±3.0 months. The population of wheezing and non-wheezing infants was homogeneous regarding weight and length at birth and at 12 months, but there was a prevalence of wheezing episodes in males (Table 1). The prevalence of occasional wheezing was 15.0%, and of recurrent wheezing, 12.7%.

Table 2 presents the clinical characteristics of wheezing infants in relation to gender. It was observed that the use of anti-leukotrienes in the treatment of wheezing, as well as the occurrence of nocturnal awakenings, were more common in male infants. The use of oral corticosteroids was common, but without significant differences between the genders. 

**Table 2:**
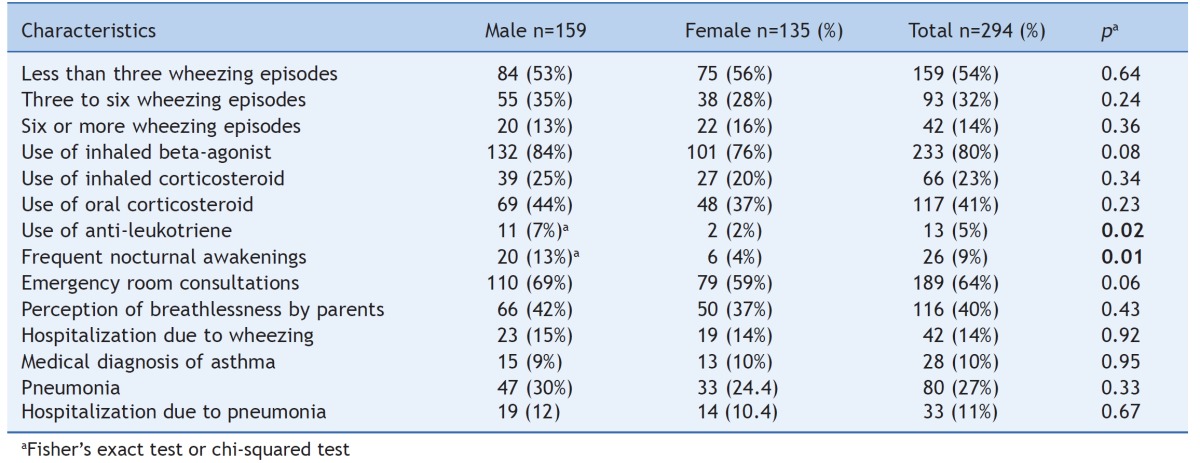
Clinical characteristics of infants with at least one wheezing episode in the first year of life according to gender (n=294).

A medical diagnosis of asthma was observed in 28 (9.5%) infants. Of the wheezing infants, 80 (27.7%) were diagnosed with pneumonia and 33 (11.2%) required hospitalization for treatment, but there were no differences regarding gender.

Table 3 presents the clinical characteristics of the infants according to the number of wheezing episodes. It was observed that the use of an inhaled bronchodilator, oral corticosteroids, and anti-leukotrienes; the perception of breathlessness by parents; frequent nocturnal awakening; hospitalization for wheezing; and medical diagnosis of asthma were more frequent in infants with recurrent wheezing. 

**Table 3:**
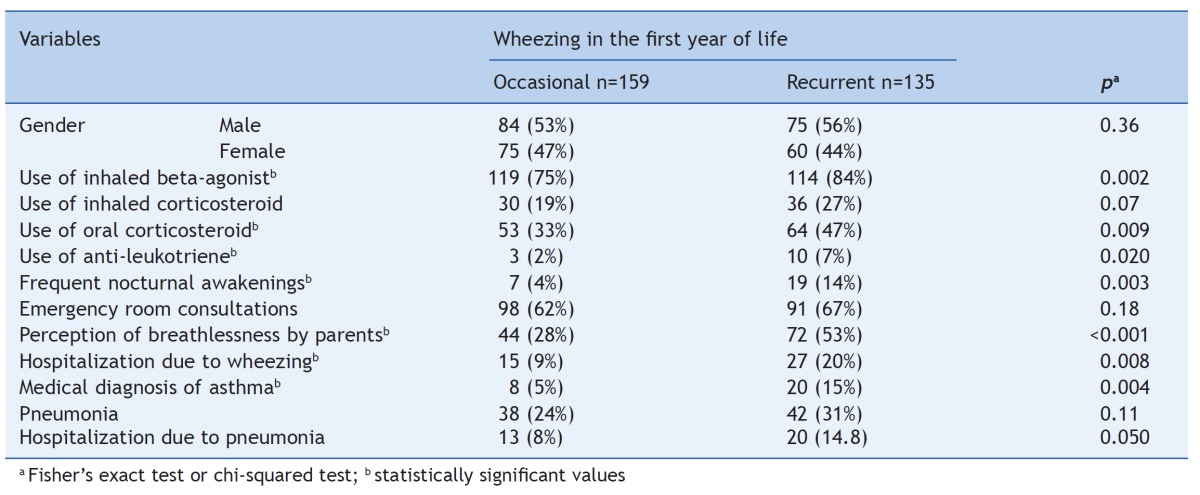
Clinical characteristics of infants according to the number of wheezing episodes (occasional [less than three episodes of wheezing] and recurrent [three or more episodes of wheezing]) in the first year of life.

At the multivariate analysis, the variables that remained associated with recurrent wheezing were the use of anti-leukotrienes (OR=4.1, 95% CI: 1.10 to 15.17) and the perception of breathlessness by parents (OR=2.9, 95% CI: 1.84 to 4.85).

## Discussion

The prevalence of occasional and recurrent wheezing in the first year of life in the city of Cuiabá was lower than those observed in other Brazilian studies, such as Curitiba, São Paulo, Recife, and Porto Alegre,^4^^-^ 7 but similar to that found in European countries.^8^ The mean prevalence of recurrent wheezing in European countries is 15.0%, while in Latin America it is 21.4%.^8^ The variability that occurs in different countries or even within the same country suggests the influence of local environmental factors on the clinical expression of several wheezing phenotypes in childhood, reinforcing the need for more studies in Brazilian cities for better comparison, as this is a country with a large territory and climate, cultural, and socioeconomic differences. 

When comparing data from Cuiabá regarding the prevalence of wheezing in the first year of life and wheezing in the last 12 months in schoolchildren and adolescents,^11^^,^ 12 a discrepancy was observed, as the values ​​are close to the observed mean national values.^13^ This finding suggests that asthma in Cuiabá may have a later onset in childhood; longitudinal studies are needed to better understand the different wheezing phenotypes in the first years of life and their association with the diagnosis of asthma in children and adolescents.

Wheezing was more common in male infants; this group showed a higher frequency of symptom severity, such as nocturnal awakenings and use of medications. Previous studies have shown that male gender is a risk factor for wheezing in childhood^6^^,^ 21 and the smaller airway-caliber in boys early in life is indicated as a possible explanation for this fact.^14^

The use of oral corticosteroids by the study infants was high, similar to that observed in other Brazilian studies.^5^^,^ 7 ^,^ 15 The use of this medication, as expected, was higher in the group of recurrent wheezing when compared to occasional wheezing, and was also higher than the use of inhaled corticosteroids. In a study of 118 infants followed-up for one year after the first wheezing episode, 37% had recurrent wheezing, even when treated with prednisolone for a short period of time. The risk for recurrent wheezing among those receiving placebo was five-fold higher in those with rhinovirus infection than in those with infection by respiratory syncytial virus (RSV). 

Among those who used prednisolone and had rhinovirus infection, there was a reduction in recurrent wheezing, a fact that was not observed in those with RSV infection. This evidences that, if there are benefits in the use of systemic corticosteroids in infants with wheezing associated with viral respiratory infections, such benefits must be related to a concomitant allergic disease.^16^ However, in preschool children with moderate virus-induced wheezing seeking emergency services, prednisolone was administered for five days aiming to verify its clinical efficacy using a symptom score, length of hospital stay, and symptom persistence. 

It was observed that there was no significant difference with the use of prednisolone in preschool children with virus-induced wheezing in relation to time of hospitalization and symptom score, even in those who had a positive asthma predictive index (API).^17^ If infants are excessively treated with oral corticosteroids, this can be explained by the fact that some centers in Brazil provide asthma medications through the public health network or because the guidelines for asthma management are not known or are inadequately followed.^18^ In Cuiabá, there is no public health program to monitor these children and parents preferentially seek emergency units, which could explain the large consumption of this type of medication in wheezing exacerbations to the detriment of preventive treatment for recurrence.

The use of anti-leukotrienes was also more frequent in the group of recurrent wheezing. International guidelines have recommended anti-leukotrienes for recurrent wheezing control therapy as an alternative to the use of inhaled corticosteroids, mainly in infants and preschool children with virus-induced wheezing.^19^^,^ 20 However, only 4.5% of wheezing infants used them in the study, which may be related to their high cost and the fact that they are not available in the public health system.

Regarding the demand for emergency services by parents, the prevalence was high (64.3%), although the rate of hospitalization for wheezing was only 14.3%, and the latter was more frequent among recurrent wheezers (OR=2.4, 95% CI: 1.22 to 4.73). The high demand for emergency services can be explained by symptom worsening in the presence of airway infections, most often of viral origin; conversely, it may be due to the fact that these emergency services are being misused as consultation sites due to failure of the primary care network in monitoring these children with recurrent wheezing.

The rate of medical diagnosis of asthma was low (9.5%), but was more frequent in the group of recurrent wheezing, a finding that is in agreement with other Brazilian studies.^4^^,^ 5 The diagnosis of asthma in this age group is very difficult, given the many wheezing phenotypes. The study of these phenotypes is extremely important to identify children with recurrent wheezing, which will have a higher risk for developing asthma over the years.^21^ In a prospective study, it was observed that most children who had wheezing in childhood had early episodes in the first year of life. Of these, half persisted with wheezing at age 6 years, being considered asthmatic.^22^ Thus, the challenge is to differentiate, among the wheezing infants, those who will persist with episodes (probable asthma) from those who are just transient wheezers.

In Brazil, the term "asthma" is often replaced by the term "bronchitis" when it comes to asthma in children, introducing an additional bias in epidemiological investigations.^23^ Even in studies that assessed the prevalence of wheezing in the past 12 months in older children (6-7 years) and adolescents (13-14 years) in two cities of Mato Grosso, the medical diagnosis of asthma was also low: 8.4% in Cuiabá and 6% in Alta Floresta.^12^^-^ 24 Due to all these factors, it can be concluded that it is difficult for physicians to differentiate between asthma in infants and recurrent wheezing disease. 

The report of pneumonia was observed in 27.2% of wheezing infants, with low hospitalization rate (11.2%) and no significant difference between the wheezing groups. In another study, both the diagnosis of pneumonia and hospitalization for pneumonia were associated with recurrent wheezing.^5^

This difference can be explained, in part, by the lower prevalence of recurrent wheezing in Cuiabá, when compared to other Brazilian studies. 

Regarding the lower airway infections caused by bacteria, such as pneumonia, it is not known whether their occurrence in the first years of life could be associated with asthma development. A recent publication showed that radiologically-confirmed bacterial pneumonia was associated with increased risk of asthma or wheezing in preschool children.^25^ Another cohort study of newborns of low socioeconomic status in Santiago (Chile) followed-up during the first year of life observed a prevalence of 13.3% of pneumonia and indicated that the presence of recurrent wheezing during the first three months of life was strongly associated with the diagnosis of pneumonia.^26^

The results of this investigation should be analyzed from a critical point of view, because, as all cross-sectional studies, it has some limitations. This study used parents' reports rather than medical reports. Evaluation of wheezing in infants is difficult for parents and may be confused with sounds coming from upper airway secretions.^27^ However, the validation study of the EISL questionnaire assessed the agreement between parents' reports and the findings of the physical examination performed by a physician, showing high agreement for most questions that employed the terms "wheezing", "wheeziness", and "bronchitis", thus demonstrating that this questionnaire is valid and reproducible to obtain reliable data on wheezing in infants aged 12-36 months of life.^28^

Moreover, the information about wheezing in the first 12 months of life was obtained when the infants were 12 to 15 months, thereby decreasing the likelihood of recall bias. Other important aspects of this study are the sample size and the implementation of a standardized questionnaire that allowed for the comparison between different centers that participated in the study. 

It can be concluded that the prevalence of wheezing in the first year of life in Cuiabá, MT, was not as high as in other Brazilian cities, but had early onset and high morbidity; however, the medical diagnosis of asthma was low. The authors emphasize the importance of implementing in Brazil a primary care program to monitor wheezing infants, aiming at the adequate management of the disease in order to reduce morbidity and improve quality of life of infants and their families.
