# Supplementary material for: Prevalence and clinical characteristics of wheezing in children in the first year of life, living in Cuiabá, Mato Grosso, Brazil
Source: Rev Paul Pediatr. 2014 Dec;32(4):313–9. doi: 10.1016/j.rpped.2014.06.004 (PMC4311784; doi:10.1016/j.rpped.2014.06.004)
Supplement: Translation

# Translation: Prevalência e características clínicas da sibilância em crianças no primeiro ano de vida, residentes na cidade de Cuiabá, Mato Grosso, Brasil☆

**Authors:** Lillian Sanchez Lacerda Moraes, Olga Akiko Takano, Javier Mallol, Dirceu Solé

**Affiliations:** a Universidade Federal de Mato Grosso (UFMT), Cuiabá, MT, Brasil; b Universidade de Santiago, Santiago, Chile; c Universidade Federal de São Paulo (UNIFESP), São Paulo, SP, Brasil

**Keywords:** Lactente, Sons respiratórios, Asma, Prevalência

## Abstract

**OBJETIVO:**

Determinar a prevalência e as características clínicas da sibilância em lactentes (12-15 meses) residentes em Cuiabá (MT).

**MÉTODOS:**

Pais e/ou responsáveis pela criança foram entrevistados e responderam ao questionário escrito padronizado do "*Estudio Internacional de Sibilancia en Lactantes*" (EISL) - fase 3, em unidades básicas de saúde por ocasião da vacinação de rotina ou durante visitas nos domicílios de crianças matriculadas nos programas de saúde da família no período de agosto de 2009 a novembro de 2010.

**RESULTADOS:**

1060 pais e/ou responsáveis responderam ao questionário escrito, sendo 514 (48,5%) lactentes do sexo masculino. Dos lactentes, 294 (27,7%) tiveram pelo menos um episódio de sibilância no primeiro ano de vida, com início aos 5,8±3,0 meses e predomínio em meninos. A prevalência de sibilância ocasional (<3 episódios de sibilância) foi 15% e a recorrente (≥3 episódios) foi 12,7%. Entre estes, o uso de broncodilatador inalado, corticosteroide oral, antileucotrieno, presença de sintomas noturnos, dificuldade para respirar e internação por sibilância foram significantemente mais frequentes. Diagnóstico médico de asma foi evidenciado em 28 (9,5%) dos lactentes sibilantes. Dos lactentes sibilantes, 80 (27,7%) relataram ocorrência prévia de pneumonia, sendo que 33 (11,2%) necessitaram internação para tratamento, porém não houve diferença entre os grupos de sibilantes.

**CONCLUSÃO:**

A prevalência de sibilância recorrente em lactentes foi mais baixa em comparação a outros estudos brasileiros, assim como o diagnóstico médico de asma. Sibilância recorrente teve início precoce e alta morbidade.

## Introdução

A sibilância é um dos sintomas respiratórios mais comuns na infância e pode se manifestar em várias doenças respiratórias, sendo a asma a mais comum. Estima-se que, aproximadamente 50-80% das crianças asmáticas desenvolvam sintomas nos primeiros cinco anos de vida, mas o diagnóstico torna-se difícil nessa faixa etária pelos obstáculos à realização de provas de função pulmonar e pela prevalência elevada de outras causas de sibilância.^1^

Apesar do grande impacto na saúde pública da sibilância recorrente na infância, principalmente em países em desenvolvimento, até recentemente não havia informações comparativas internacionais sobre a prevalência de sibilância obtida por instrumento padronizado e validado, sobretudo no primeiro ano de vida, quando as crianças são mais vulneráveis às complicações decorrentes de características anatômicas, funcionais e imunológicas das vias aéreas.^2^

Para avaliar o impacto da sibilância recorrente em lactentes e determinar sua prevalência e fatores de risco a ela associados, foi desenvolvido o *Estudio Internacional de Sibilancias en Lactantes* (EISL) no primeiro ano de vida. Trata-se de um estudo multicêntrico internacional do qual participaram países da América Latina, Espanha e Holanda.^3^ Os primeiros dados obtidos no Brasil, com o EISL, foram em Curitiba em 3.003 lactentes e observou-se que, nos primeiros 12 meses de vida, 45,4% tiveram pelo menos um episódio de sibilância e 22,6% tiveram sibilância recorrente (três ou mais episódios), denotando alta prevalência de sibilância, com início precoce e alta morbidade.^4^ Em São Paulo, dentre 1.014 lactentes residentes na região centro-sul, 46% manifestaram pelo menos um episódio de sibilância e 26,6% sibilância recorrente. Ainda em São Paulo, o início da sibilância foi precoce, por volta dos cinco meses de vida, e a proporção de lactentes diagnosticados e tratados como asmáticos foi baixa, o que demonstra a dificuldade do diagnóstico médico de asma nesta faixa etária.^5^ A prevalência de pelo menos um episódio de sibilância no primeiro ano de vida foi de 61% dos lactentes residentes na cidade de Porto Alegre, RS,^6^ e de 43% dos lactentes na cidade de Recife, PE.^7^

A compilação de dados da primeira fase do estudo realizado em países da América Latina e Europa observou que, entre 30.093 lactentes, 45,2% tiveram pelo menos um episódio de sibilância e 20,3% sibilância recorrente, sendo que a prevalência média de sibilância recorrente em países da América Latina foi 21,4%, enquanto nos países da Europa foi de 15%. Houve morbidade significante associada com sibilância recorrente em termos de episódios graves, idas à emergência, admissões hospitalares e uso de corticosteroides inalatórios.^8^

Considerando-se ser a sibilância em lactentes um sintoma muito frequente, cuja prevalência varia nos diferentes centros, mas as causas para tais variações ainda estão em investigação, e que que Brasil é um país de grande extensão territorial, com diferenças climáticas, culturais e socioeconômicas nas diversas regiões, o objetivo deste estudo foi verificar a prevalência e características clínicas da sibilância em lactentes residentes em Cuiabá (MT), utilizando o instrumento padronizado do EISL - fase 3. 

## Método

Participaram deste estudo pais ou responsáveis de lactentes com idade entre 12 e 15 meses, residentes no município de Cuiabá e que responderam o questionário escrito padrão do EISL (QE-EISL) - fase 3. Pais e/ou responsáveis foram convidados a participar do estudo quando da sua ida a Unidades Básicas de Saúde (UBS) para consulta e/ou vacinação de rotina de seus filhos ou por meios de visita nos domicílios de crianças matriculadas nos Programas de Saúde da Família das UBS. Esta visita era realizada junto com as agentes de saúde para facilitar o acesso aos domicílios.

Na época da realização do estudo, existiam 60 Unidades Básicas de Saúde (UBS) distribuídas em quatro regiões (Norte, Sul, Leste e Oeste). Nas regiões norte, oeste e leste a densidade demográfica é médio-alta: 57,4-86,0 habitantes/hectare (hab/ha) e, na região sul, é médio-baixa (11,05-28,76 hab/há). Foram selecionadas por sorteio para aplicação dos questionários escritos 28 UBS, sendo seis unidades nas regiões norte, oeste e leste e 10 unidades na região sul, devido a menor densidade populacional. As visitas às UBS ocorreram entre agosto de 2009 e novembro de 2010 e durante as duas campanhas de imunização contra poliomielite infantil realizadas neste período, sendo que todas as crianças da faixa etária pretendida que compareceram no dia da campanha participaram do estudo. Pais e/ou responsáveis assinaram o termo de consentimento livre e esclarecido (TCLE) e, em seguida, foram entrevistados pela pesquisadora principal ou um estudante de Medicina da Universidade Federal de Mato Grosso (UFMT) previamente treinado.

O QE-EISL fase 3 é um instrumento formado por 50 perguntas sobre características demográficas, sibilância e fatores de risco, traduzido para o Português e validado para nossa população.^9^ Para a realização do estudo, foi estipulado pelos coordenadores do EISL que a amostra deveria incluir pelo menos 1.000 lactentes. O tamanho amostral foi baseado no *International Study of Asthma and Allergies in Childhood *(ISAAC), considerando uma prevalência de sibilância de 30% e 25% em dois centros diferentes, com força de estudo de 95% e nível de significância de 1% para esta amostra, a fim de garantir poder adequado às comparações entre centros e países, mesmo para perguntas com baixa prevalência de respostas afirmativas.^3^^,^ 10

Os dados obtidos foram codificados de forma padrão, transferidos a um banco de dados elaborados no Microsoft Excel^(r)^ 2007 e analisados estatisticamente com a utilização do SPSS for Windows - versão 18.0. Os lactentes sibilantes foram separados em grupos levando-se em consideração a frequência da sibilância: menos de três episódios de sibilância (sibilantes ocasionais) e três ou mais episódios de sibilância (sibilantes recorrentes)**. **Os que nunca apresentaram sibilância foram denominados não sibilantes.

Na dependência das variáveis analisadas, foram empregados testes paramétricos (teste t de Student) e não paramétricos (Qui-quadrado, teste exato de Fisher) e regressão logística para análise conjunta dos possíveis fatores de gravidade relacionados à sibilância recorrente. Considerou-se o nível de significância de 0,05 para rejeição da hipótese de nulidade.

Este trabalho foi aprovado pelos Comitês de Ética em Pesquisa da Universidade Federal de São Paulo/Escola Paulista de Medicina - UNIFESP/EPM e pelo Comitê de Ética em Pesquisa do Hospital Universitário Júlio Müller/UFMT em Cuiabá-MT.

## Resultados

Foram entrevistados 1.060 pais e/ou responsáveis de lactentes entre 12 e 15 meses de vida. Nenhum questionário foi excluído da amostra por preenchimento incorreto. A maioria dos respondedores foram mães (87,9%), seguidos de outros familiares (6,4%) e pais (5,7%). Eram do sexo feminino 546 (51,5%) crianças. As características gerais da população estudada encontram-se na tabela 1.

**Tabela 1:** Características gerais da população avaliada (n=1.060)

Dos lactentes analisados, 294 (27,7%) tiveram pelo menos um episódio de sibilância no primeiro ano de vida (sibilantes), com início da sibilância aos 5,8±3,0 meses. A população de lactentes sibilantes e não sibilantes foi homogênea quanto ao peso e estatura ao nascimento e aos 12 meses, porém houve predomínio de sibilância no sexo masculino (tabela 1). A prevalência de sibilância ocasional foi 15,0% e a de recorrente foi 12,7% na população estudada.

A tabela 2 apresenta as características clínicas dos lactentes sibilantes em relação ao sexo. Observou-se que o uso de antileucotrieno no tratamento da sibilância e a ocorrência de despertar noturno foram mais frequentes entre lactentes do sexo masculino. O uso de corticosteroide oral foi frequente, mas sem diferenças significantes entre os sexos.

**Tabela 2:** Características clínicas dos lactentes que sibilaram pelo menos uma vez no primeiro ano de vida discriminados quanto ao sexo (n=294)

Diagnóstico médico de asma foi observado em 28 (9,5%) dos lactentes. Dos lactentes sibilantes, 80 (27,7%) tiveram diagnóstico de pneumonia, sendo que 33 (11,2%) necessitaram internação para tratamento, porém não houve diferença em relação ao sexo. 

A tabela 3 mostra as características clínicas dos lactentes segundo o número de episódios de sibilância. Observou-se que o uso de broncodilatador inalatório, corticosteroide oral e antileucotrieno, a percepção de falta de ar pelos pais, despertar noturno frequente, hospitalização por sibilância e diagnóstico médico de asma foram mais frequentes nos lactentes com sibilância recorrente.

**Tabela 3:** Características clínicas dos lactentes segundo o número de episódios de sibilância: ocasional (menos de três episódios de sibilância) e recorrente (três ou mais episódios de sibilância) no primeiro ano de vida

Na análise multivariada, as variáveis que se mantiveram associadas à sibilância recorrente foram o uso de antileucotrieno (OR=4,1; IC95%=1,10-15,17) e a percepção de falta de ar pelos pais (OR=2,9; IC95%=1,84-4,85).

## Discussão

A prevalência de sibilância ocasional e recorrente no primeiro ano de vida na cidade de Cuiabá foi mais baixa do que as observadas em outros estudos brasileiros como Curitiba, São Paulo, Recife e Porto Alegre,^4^^-^ 7 porém semelhante à encontrada em países da Europa.^8^ A prevalência média de sibilância recorrente em países europeus foi 15,0%, enquanto que nos países da América Latina foi 21,4%.^8^ A variabilidade que ocorre em diferentes países ou até mesmo dentro de um mesmo país sugere a influência de fatores locais ambientais na expressão clínica dos vários fenótipos de sibilância na infância, reforçando a necessidade de estudos em mais cidades do Brasil para melhor comparação, uma vez que este é um país de grande extensão territorial com diferenças climáticas, culturais e socioeconômicas.

Ao comparar os dados de Cuiabá com relação à prevalência de sibilância no primeiro ano de vida em lactentes e os de sibilância nos últimos 12 meses em escolares e adolescentes,^11^^,^ 12 verifica-se discrepância, posto que os valores se aproximam da média nacional observada.^13^ Este achado sugere que a asma em Cuiabá pode ter início mais tardio na infância e estudos longitudinais são necessários para melhor entender os diferentes fenótipos de sibilância nos primeiros anos de vida e sua associação com o diagnóstico de asma em crianças e adolescentes.

A sibilância foi mais frequente nos lactentes do sexo masculino e estes apresentaram maior frequência de sintomas de gravidade como despertar noturno e utilização de medicamentos. Estudos anteriores já demonstraram que o sexo masculino é fator de risco para sibilância na infância,^6^^,^ 21 apontando-se o menor calibre das vias aéreas dos meninos nos primeiros anos de vida como uma possível explicação para esse fato.^14^

O consumo de corticosteroide oral pelos lactentes estudados foi elevado, semelhante ao observado em outros estudos brasileiros.^5^^,^ 7 ^,^ 15 A utilização deste medicamento, como seria esperada, foi maior no grupo de sibilantes recorrentes quando comparados aos ocasionais e também foi maior que o uso de corticosteroide inalatório. Em um estudo com 118 lactentes seguidos por um ano após o primeiro episódio de sibilância, 37% apresentaram sibilância recorrente mesmo quando tratados com prednisolona por um curto período. O risco para sibilância recorrente entre os que utilizaram placebo foi cinco vezes maior naqueles que apresentavam infeccção por rinovírus do que aqueles com infecção por vírus sincicial respiratório (VSR). Entre os que usaram prednisolona e tiveram infecção por rinovírus, houve redução da sibilância recorrente, fato não observado naqueles com infecção por VSR. Isso mostra que, se há benefícios no uso de corticosteroides sistêmicos em lactentes com sibilância associada à infecção respiratória viral, tal benefício deve estar relacionado à concomitância de doença alérgica.^16^ Entretanto, em pré-escolares com sibilância moderada induzida por vírus que procuravam serviços de emergência, foi administrada prednisolona por cinco dias com o objetivo de verificar a sua eficácia clínica por meio de um escore de sintomas, do tempo de hospitalização e da persistência dos sintomas. Observou-se não haver diferença significativa com o uso de prednisolona nos pré-escolares com sibilância por vírus em relação ao tempo de hospitalização e ao escore de sintomas, mesmo naqueles que apresentaram um índice preditivo positivo para asma (IPA).^17^ Se os lactentes são tratados excessivamente com corticosteroide oral isto pode ser explicado pelo fato de que alguns centros no Brasil disponibilizam medicações para asma na rede pública ou porque as diretrizes para o manejo da asma não são conhecidas ou são inadequadamente observadas.^18^ Em Cuiabá, não há um programa de saúde pública para acompanhamento destas crianças e os pais procuram preferencialmente as unidades de emergência, o que justificaria o grande consumo deste medicamento nas exacerbações de sibilância, em detrimento ao tratamento preventivo de sua recorrência.

O uso de antileucotrieno também foi mais frequente no grupo de sibilantes recorrentes. Consensos internacionais têm recomendado os antileucotrienos na terapia de controle de sibilância recorrente, como alternativa ao uso de corticosteroides inalados, particularmente em lactentes e pré-escolares com sibilância induzida por vírus.^19^^,^ 20 Porém, apenas 4,5% dos lactentes sibilantes o utilizaram no estudo, o que pode estar relacionado ao seu alto custo e por não estar disponibilizado na rede pública de saúde.

Em relação à procura dos serviços de emergência pelos pais, a prevalência foi elevada (64,3%), apesar disto a internação por crise de sibilância foi de apenas 14,3% e esta última foi mais frequente entre os sibilantes recorrentes (OR=2,4; IC95%=1,22-4,73). A grande procura pelos serviços de emergência pode ter como explicação o agravamento dos sintomas diante de infecções de vias aéreas, na maior parte das vezes de origem viral, mas, por outro lado, pode se dever ao fato de que estes locais estão sendo indevidamente utilizados como locais de consultas por falha na rede de atendimento básico no acompanhamento destas crianças com sibilância recorrente.

O diagnóstico médico de asma foi baixo (9,5%), porém foi mais frequente no grupo de sibilantes recorrentes, em concordância com outros estudos brasileiros.^4^^,^ 5 O diagnóstico de asma nessa faixa etária é muito difícil em face dos inúmeros fenótipos de sibilância. O estudo destes fenótipos tem sido de extrema importância na identificação de crianças com sibilância recorrente, que terão maior risco para o desenvolvimento de asma no decorrer dos anos.^21^ Em estudo prospectivo, observou-se que a maioria das crianças que apresentaram sibilância na infância manifestaram os primeiros episódios no primeiro ano de vida. Dessas, metade persistiu com sibilância até os seis anos de idade, sendo considerada asmática;^22^ portanto o desafio é distinguir, dentre os lactentes sibilantes, aqueles que persistirão sibilando, os prováveis asmáticos, daqueles que são apenas sibilantes transitórios. 

No Brasil, o termo "asma" é frequentemente substituído pelo termo "bronquite", quando diz respeito à asma em crianças, introduzindo um viés adicional nas investigações epidemiológicas.^23^ Mesmo em estudos que avaliaram a prevalência de sibilância nos últimos 12 meses em crianças maiores (6-7 anos) e adolescentes (13-14 anos) em duas cidades de Mato Grosso, o diagnóstico médico de asma também foi baixo, sendo 8,4% em Cuiabá e 6% em Alta Floresta.^12^^-^ 24 Por todos estes fatores, conclui-se que há uma grande dificuldade por parte do médico em diferenciar asma em lactentes com doença sibilante recorrente.

O relato de pneumonia foi observado em 27,2% dos lactentes sibilantes, com baixa taxa de hospitalização (11,2%) e não houve diferença significante entre os grupos de sibilantes. Em outro estudo, tanto o diagnóstico de pneumonia como a hospitalização por pneumonia estiveram associados com sibilância recorrente.^5^ Esta diferença pode ser explicada, em parte, devido à menor prevalência de sibilância recorrente em Cuiabá, quando comparada a outros estudos brasileiros.

Com relação às infecções de vias aéreas inferiores causadas por bactérias como a pneumonia, ainda não se sabe se a sua ocorrência nos primeiros anos de vida poderia estar associada ao desenvolvimento de asma. Publicação recente demonstrou que pneumonia bacteriana confirmada radiologicamente foi associada a risco maior de asma ou sibilância em pré-escolares.^25^ Outro estudo de coorte de recém-nascidos de baixo nível socioeconômico em Santiago (Chile) acompanhados durante o primeiro ano de vida indicou prevalência de pneumonia de 13,3% e a presença de sibilância recorrente durante os três primeiros meses de vida esteve fortemente associada ao diagnóstico de pneumonia.^26^

Os resultados da presente investigação devem ser analisados do ponto de vista crítico, pois, como todo estudo transversal, tem suas limitações. Este estudo utiliza-se de relato dos pais e não de relato médico. A avaliação da sibilância em lactentes é difícil para os pais, podendo ser confundida com sons oriundos de secreções de vias aéreas superiores.^27^ Porém, estudo de validação do questionário do EISL avaliou a análise de concordância entre o relato dos pais e o achado de exame físico realizado por médico, mostrando alta concordância para a maioria das perguntas que emprega os termos "chiado no peito", "sibilância" e "bronquite", demonstrando que este questionário é válido e reprodutível na obtenção de dados confiáveis sobre sibilância em lactentes de 12-36 meses de vida.^28^ Além disso, a obtenção das informações sobre a sibilância ocorrida nos primeiros 12 meses de vida foi realizada quando os lactentes estavam com 12 a 15 meses, diminuindo assim a probabilidade de viés de memória. Outros aspectos importantes deste estudo são o tamanho da amostra e a aplicação de um questionário padronizado que possibilitou a comparação entre os diferentes centros que realizaram o estudo.

Concluímos que a prevalência de sibilância no primeiro ano de vida em Cuiabá, MT, não foi tão elevada quanto nos outros centros brasileiros, mas teve início precoce e alta morbidade, mas, apesar disso, o diagnóstico médico de asma foi baixo. Ressalta-se a importância de implantação, em nosso meio, de um programa de atenção básica para acompanhamento de lactentes sibilantes, visando o manejo adequado da doença no intuito de diminuir sua morbidade e melhorar a qualidade de vida de lactentes e seus familiares.
